# Characteristics of patients assessed for cognitive decline in primary healthcare, compared to patients assessed in specialist healthcare

**DOI:** 10.1080/02813432.2020.1753334

**Published:** 2020-05-02

**Authors:** Mona Michelet, Anne Lund, Bjørn Heine Strand, Knut Engedal, Geir Selbaek, Sverre Bergh

**Affiliations:** a Norwegian National Advisory Unit on Ageing and Health, Vestfold Hospital Trust, Tønsberg, Norway;; b Department of Geriatric Medicine, Oslo University Hospital, Oslo, Norway;; c Faculty of Medicine, University of Oslo, Oslo, Norway;; d Faculty of Health Sciences, Department of Occupational Therapy, Prosthetics and Orthotics, OsloMet – Oslo Metropolitan University, Oslo, Norway;; e Norwegian Institute of Public Health, Oslo, Norway;; f Centre for Old Age Psychiatric Research, Innlandet Hospital Trust, Ottestad, Norway

**Keywords:** Dementia, diagnostic services, depression, primary health care, activities of daily living

## Abstract

**Objective:** The aim of this study was to describe patients assessed for cognitive decline in primary healthcare, compared to patients assessed in specialist healthcare and to examine factors associated with depression.

**Design:** This was an observational study.

**Setting:** Fourteen outpatient clinics and 33 general practitioners and municipality memory teams across Norway.

**Subjects:** A total of 226 patients assessed in primary healthcare and 1595 patients assessed in specialist healthcare outpatient clinics.

**Main outcome measures:** Cornell scale for depression in dementia (CSDD), Mini-Mental Status Examination (MMSE), Clock drawing test, Informant Questionnaire on Cognitive Decline in the Elderly (IQCODE), Instrumental Activities of Daily Living, Personal Self-Maintenance Scale, Relatives’ stress scale (RSS), and Neuropsychiatric Inventory Questionnaire (NPI-Q)

**Results:** Patients assessed in primary healthcare were older (mean age 81.3 vs 73.0 years), less educated, had poorer cognition (MMSE median 22 vs 25), more limitations in activities of daily living (ADL), more behavioural and psychological symptoms of dementia (BPSD), more depressive symptoms (CSDD median 7 vs 5), more often lived alone (60% vs 41%) and were more often diagnosed with dementia (86% vs 47%) compared to patients diagnosed in specialist healthcare. Depression was associated with female gender, older age, more severe decline in cognitive functioning (IQCODE, OR 1.65), higher caregiver burden (RSS, OR 1.10) and with being assessed in primary healthcare (OR 1.53).

**Conclusion:** Post-diagnostic support tailored to patients diagnosed with dementia in primary healthcare should consider their poor cognitive function and limitations in ADL and that these people often live alone, have BPSD and depression.

**Key points**
People diagnosed in Norwegian primary healthcare had more needs than people diagnosed in specialist healthcare.
  • They were older, less educated, had poorer cognitive functioning and activity limitations, more often lived alone, and had more BPSD and depression.
  • Depression was associated with being female, older, having cognitive decline, being assessed in primary care and the caregiver experiencing burden
  • Post diagnostic support for people with dementia should be tailored to the individual’s symptoms and needs.

## Introduction

Globally, the number of people with dementia was estimated to be 35.6 million in 2010, a number expected to double every 20 years [1]. Thorough assessment and diagnosis are keys to providing effective medical treatment and individually tailored support for people with dementia. However, many with dementia are not assessed or given a timely diagnosis, and the rate of undetected dementia varies between 31% and 96% with a pooled rate of 62% [2]. Common unmet needs of people with dementia involve daytime activities, social companionship, and psychological needs [3]; thus, facilitating participation in meaningful activities may improve well-being in this population [4].

Depression is common in people with dementia [5] and may lead to negative outcomes including reduced quality of life, disability in activities of daily living (ADL), and a more rapid development of cognitive decline [6]. Therefore, assessing people with cognitive decline for symptoms of depression and targeting support when symptoms are present are important.

In Norway, assessing and diagnosing people over 65 years old with symptoms of cognitive decline is mainly a primary healthcare responsibility [7,8] and performed by general practitioners (GPs), usually in collaboration with a community-based multidisciplinary memory team, found in approximately 90% of municipalities. The teams also play a central role in post-diagnostic support for home-dwelling people with dementia. Those under 65 years with symptoms of cognitive decline, as well as older patients presenting complicated or unclear symptoms or severe behavioural and psychological symptoms of dementia (BPSD) should be referred to a specialist healthcare service [9]. The Norwegian national guideline on dementia recommends using a standardised basic diagnostic protocol in primary healthcare and a standardised comprehensive diagnostic protocol in specialist healthcare [9].

A Swedish study comparing patients diagnosed in specialist and primary healthcare found that primary healthcare patients were older, had more severe cognitive decline, and were more likely to receive in-home care or day care [10]. A UK study evaluating a primary healthcare dementia diagnostic service found that patients and caregivers generally experienced high-quality diagnostic service in primary care [11].

There is an ongoing discussion in Norway about whether GPs are fulfilling their role in diagnosing dementia. More knowledge about people assessed in primary healthcare may contribute to this debate and provide a better basis for recommending how assessing and diagnosing people with cognitive decline should be organised in the future. Such knowledge is also important for providing individually tailored post-diagnostic support to home-dwelling people with dementia.

Thus, the main aim of this study was to describe patients assessed for cognitive decline in primary healthcare compared to those assessed in specialist healthcare. As depression is common in dementia and may complicate the presentation of the symptoms, we also wanted to explore depressive symptomatology in patients and examine factors, including place of assessment, associated with depression.

## Material and methods

### Participants

#### Primary healthcare cohort (PrimCare)

In all, 226 home-dwelling patients with cognitive decline were recruited in 2013 and 2014. Data were collected by experienced memory teams from a convenience sample of 33 of a total of 428 Norwegian municipalities. The only inclusion criteria were a referral to a memory team by their GP and consenting to participate. There were no exclusion criteria.

#### Specialist healthcare cohort (SpecCare)

In all, 1,595 home-dwelling patients with cognitive decline were recruited from 14 outpatient clinics across Norway. All had been included in the Norwegian register of persons assessed for cognitive symptoms (NorCog), a consent-based quality and research register. There were no exclusion criteria. To ensure that no patients would appear in both cohorts, NorCog data from 2011 and 2012 were used. The NorCog register recruits patients from memory clinics, geriatric clinics and old-age psychiatry clinics. Memory clinics primarily assess patients with suspected neurodegenerative diseases and represent a type of highly specialised multidisciplinary clinic. The two latter types of clinics also assess patients with other diseases and differ from the memory clinics regarding demographic characteristics [12]. To compare participants from different types of outpatient clinics with participants from primary healthcare, the outpatient clinics were dichotomised into memory clinics and ‘other’ clinics (geriatric and old-age psychiatry outpatient clinics).

### Assessment measures and diagnostic procedures

#### Measures

At the assessment the patients were accompanied by a next of kin, and the following measures, included in the diagnostic protocol both in primary and specialist healthcare, were used in the study:

Tests: the Norwegian revised version of the Mini Mental State Examination (MMSE-NR2) with scores ranging from zero to 30 and a higher score indicating better cognitive performance [13], the clock-drawing test (CDT) with scores zero to five and a higher score indicating better cognitive performance, dichotomised with a cut-off of 3/4 [14].

Proxy-based measures: The Informant Questionnaire of Cognitive Decline in the Elderly (IQCODE), measuring change in cognition compared to ten years earlier and providing an average score ranging from one to five where a score above 3.44 indicates a significant decline in cognitive function [15], the Instrumental Activities of Daily Living (IADL) scale ranging from one to eight with a lower score indicating a higher level of dependence [16], the Physical Self-Maintenance Scale (PSMS) ranging from one to six with a lower score indicating a higher level of dependence [16], the Cornell Scale for Depression in Dementia (CSDD) ranging from zero to 38 and a higher score indicating more depressive symptoms [17], and the Neuropsychiatric Inventory-Questionnaire (NPI-Q) addressing the severity of 12 neuropsychiatric symptoms, each on a scale from one to three with three indicating more severe symptoms [18]. In addition, carers completed the Relatives’ Stress Scale (RSS) ranging from zero to 60, with higher scores indicating a higher level of carer burden [19].

#### Diagnoses

PrimCare patients were given an ICPC-2 diagnosis by their GP [20]. Additionally, for the purpose of this study, they were given research diagnoses by two experienced psychiatrists in consensus based on all available information: 1) no dementia/other diseases, 2) subjective cognitive impairment (SCI), 3) mild cognitive impairment (MCI) and 4) dementia. Dementia was diagnosed using ICD-10 criteria for research [21] and MCI was diagnosed using the Winblad criteria [22]. SCI was used when the person had a subjective experience of cognitive decline but normal cognitive test results (MMSE and CDT).

In specialist healthcare in Norway, ICD-10 criteria for diagnoses are used. In the SpecCare cohort, the criteria for research diagnosis of dementia, MCI, and SCI were the same as in the PrimCare cohort. As information collected in PrimCare was insufficient to establish aetiological diagnoses, none were retrieved from SpecCare either.

Seven patients were excluded from the SpecCare cohort because the researchers found discrepancies between the collected data and the clinical diagnosis.

### Missing data

A total of 4% of participants in the PrimCare cohort and 10% in the SpecCare cohort had missing data on all proxy-based measures.

Missing data imputation within scales was done for participants with a maximum of 50% of items missing on an individual scale using the expectation–maximisation imputation method. Parallel to this, a copy of the dataset was prepared, imputing subject mean on scales with a maximum 20% of missing items. To quality check the imputation done with the expectation–maximisation method, the main analyses were also performed in the file imputed with subject mean, and the results of these secondary analyses were comparable to those presented in this manuscript, with similar trends for *p*-values and odds ratios (secondary analyses not presented).

### Statistics

Initially, to group symptoms and reduce the number of variables, a principal component analysis was performed on the items of the NPI-Q scale, in line with Trzepacz *et al* [23]. We used Varimax rotation and an eigenvalue greater than 1, resulting in the following three components used in the analyses: (i) psychosis symptoms (delusions and hallucinations); (ii) affective symptoms (depression/dysphoria, anxiety, appetite/eating, night-time behaviours, apathy/indifference, and motor disturbance); and iii) agitation symptoms (agitation/aggression, disinhibition, irritability/lability and elation/euphoria).

To compare the PrimCare cohort with the SpecCare cohort (the latter as one group and dichotomised into memory clinics and ‘other’ clinics), we used descriptive analyses with *t*-tests or Mann–Whitney *U* tests for continuous variables and the chi-square test for categorical variables. Since age, according to the national guideline, is the main criterion for place of assessment, we analysed whether any differences between the cohorts remained when adjusting for age, using binary and multinomial logistic analyses.

Binary logistic regression was performed to examine factors associated with depression, and we used the CSDD as a measure of depression. CSDD scores were dichotomised using a 5/6 cut-off, found to be valid in a previous Norwegian study of home-dwelling people with cognitive decline assessed in memory clinics [5]. This method was preferred over linear regression due to a highly skewed distribution on CSDD. Selection of independent variables was done considering a combination of clinical, theoretical, and statistical factors. Only participants with data on all the selected variables were included in the regression analysis, 174 from the PrimCare cohort and 975 from the SpecCare cohort. Variables were entered in the model in steps predefined by the authors.

Data were analysed using IBM SPSS Statistics version 25.0.

### Ethics

NorCog has permission from the Norwegian Data Protection Authority to collect data until 2029. The PrimCare project was approved by the ethics committee for medical research in South-East Norway with reference number 2012/1997. All participants and participating relatives in both cohorts signed informed consent. Data from the two cohorts were completely anonymised before being merged into one datafile for analyses, which was confirmed by the Norwegian Centre for Research Data to be in accordance with the regulations.

## Results

### Diagnoses

In all, 52 patients did not receive a diagnosis from their GPs. Of the 174 patients who did, agreement between the GPs’ diagnoses and the research diagnoses made by the two experts for the purpose of the study was found in 144 (82%) cases.

### Characteristics of patients in PrimCare compared to SpecCare

Compared to the total SpecCare cohort, the PrimCare cohort were older and less educated; had poorer cognition as indicated by scores on the MMSE-NR, CDT, and IQCODE; had more limitations in ADL as indicated by the PSMS and IADL; experienced more neuropsychiatric symptoms as indicated by the NPI-Q, and more symptoms of depression as indicated by the CSDD. A larger proportion of the patients lived alone and were diagnosed with dementia (Table 1).

**Table 1.** Comparison between patients assessed in primary healthcare and patients assessed in specialist healthcare; the latter as one group and as two sub-groups.

Variable	Primary healthcare *n* = 226	Specialist healthcare					
		All *n* = 1595	*p* Value^1^	Geriatric and old-age psychiatry clinics *n* = 967	*p* Value^2^	Memory clinics *n* = 628	*p* Value^3^
Gender – % women	59.7	55.1	0.216	58.3	0.755	50.2	0.017
Age patient – mean (SD)	81.3 (6.7)	73.0 (10.6)	<0.001	76.2 (9.1)	<0.001	67.9 (10.8)	<0.001
Age relative – mean (SD)	63.1 (13.5)	61.3 (14.1)	0.087	62.1 (14.2)	0.330	60.5 (13.9)	0.018
	*n* = 210	*n* = 1121		*n* = 621		*n* = 500	
Education, years – median (Q1, Q3)	8.5 (7, 11)	11.0 (8, 14)	<0.001	10 (8, 13)	<0.001	12 (9, 15)	<0.001
	*n* = 222	*n* = 1465		*n* = 858		*n* = 607	
% living with someone	40.2	59.2	<0.001	57.5	<0.001	61.7	<0.001
	*n* = 224	*n* = 1519		*n* = 916		*n* = 603	
Diagnosis – %							
SCI/ not dementia	3.5	21.6		13.7		33.8	
MCI	10.6	31.8	<0.001	33.7	<0.001	28.8	<0.001
Dementia	85.8	46.6		52.6		37.4	
MMSE, sumscore – median (Q1, Q3)	22.0 (19, 25)	25.0 (21, 28)	<0.001	24 (20, 27)	<0.001	26 (23, 28)	<0.001
	*n* = 223	*n* = 1565		*n* = 951		*n* = 614	
Clock drawing test – % score 4 or 5	33.0	55.1	<0.001	47.2	<0.001	67.2	<0.001
	*n* = 218	*n* = 1538		*n* = 931		*n* = 607	
IQCODE score – mean (SD)	4.15 (0.49)	3.83 (0.57)	<0.001	3.91 (0.59)	<0.001	3.68 (0.53)	<0.001
	*n* = 213	*n* = 1395		*n* = 863		*n* = 532	
PSMS – median (Q1, Q3)	4 (3, 5)	5 (4, 6)	<0.001	5 (4, 6)	<0.001	6 (5, 6)	<0.001
	*n* = 214	*n* = 1344		*n* = 826		*n* = 518	
IADL – median (Q1, Q3)	5 (3, 6)	6 (4, 7)	<0.001	5 (4, 7)	<0.001	7 (5, 8)	<0.001
	*n* = 186	*n* = 1175		*n* = 709		*n* = 466	
CSDD – median (Q1, Q3)	7 (3, 12)	5 (2, 10)	0.001	5 (2, 11)	0.001	5 (3, 9)	0.001
	*n* = 191	*n* = 1281		*n* = 772		*n* = 509	
NPI-Q – median (Q1, Q3)							
Psychosis symptoms	0 (0, 1)	0 (0, 0)	<0.001	0 (0, 1)	<0.001	0 (0, 0)	<0.001
Affective symptoms	4 (1, 5)	3 (1, 5)	0.023	3 (1, 5)	0.039	2 (1, 6)	0.021
Agitation symptoms	1 (0, 3)	1 (0, 2)	0.047	1 (0, 2)	0.067	0 (0, 2)	0.048
	*n* = 156*	*n* = 1337*		*n* = 827*		*n* = 510*	
RSS – median (Q1, Q3)	11 (6, 22.75)	10 (4, 21)	0.069	11 (4, 23)	0.415	9 (3, 18.75)	0.002
	*n* = 204	*n* = 1294		*n* = 798		*n* = 496	

SD: standard deviation; Q: quartile; SCI: subjective cognitive impairment; MCI: mild cognitive impairment; MMSE: mini mental status examination; IQCODE: informant questionnaire on cognitive decline in the elderly – mean score of 16 items; PSMS: Physical Self Maintenance Scale; IADL: Instrumental Activities of Daily Living; CSDD: Cornell Scale for Depression in Dementia; NPI-Q: Neuropsychiatric Inventory-Questionnaire; RSS: Relatives’ Stress scale. ^1^*p*-value from *t*-tests; Mann–Whitney *U* tests or chi-square tests; for difference PrimCare vs SpecCare all. ^2^*p*-value from *t*-tests; Mann–Whitney *U* tests or chi-square tests; for difference PrimCare vs SpecCare ‘other’. ^3^*p*-value from *t*-tests; Mann–Whitney *U* tests or chi-square tests; for difference PrimCare vs SpecCare memory clinic.

*N is different for the three subsyndromes of NPI-Q; this is the lowest *n*.

#### Characteristics of the PrimCare cohort compared to the SpecCare memory clinics cohort

There was a larger proportion of women in the PrimCare cohort than in the memory clinic cohort; PrimCare relatives were older and reported higher caregiver burden; and PrimCare patients had significantly more symptoms on all three NPI-Q domains (psychosis, affective symptoms, and agitation).

#### Characteristics of the PrimCare cohort compared to SpecCare ‘other’ cohort

PrimCare patients had more psychotic and affective symptoms (NPI-Q), but not more agitation as compared to SpecCare patients.

Even though the differences between the PrimCare cohort and both cohorts within SpecCare were significant, the mean/median scores indicate that the PrimCare cohort was more similar to the ‘other’ SpecCare cohort than to the memory clinic cohort (Table 1).

#### Characteristics adjusted for age

Overall, results were somewhat attenuated when adjusting for age, but no significant changes were observed for most characteristics (see Tables 2 and 3). However, the difference between the PrimCare cohort and the total SpecCare cohort regarding scores on the NPI-Q affective and agitation subsyndromes became significant when adjusting for age, with more severe symptoms in the PrimCare cohort. Further, the OR for ‘living with someone’ versus ‘living alone’ did not remain significant between the PrimCare cohort and the SpecCare memory clinic cohort, even though the OR was in the same direction (Crude model: OR = 2.40, 95% CI 1.75, 3.28; age-adjusted model: OR = 1.35, 95% CI 0.95, 1.91). Gender differed between the PrimCare cohort and the memory clinic cohort in unadjusted analyses but not when adjusting for age.

**Table 2.** Odds ratios of being assessed for cognitive decline in specialist healthcare (SpecCare – all) versus primary healthcare (PrimCare) by background factors, diagnoses and scores on cognitive and functional tests.

Primary healthcar*e* : 0 Specialist healthcare = 1	Unadjusted			Adjusted for age		
	OR	95% CI	*p* Value	OR	95% CI	*p* Value
Gender (female = Ref) *n* = 226/1595	1.21	0.91, 1.61	0.191	0.98	0.73, 1.32	0.882
Age patient *n* = 226/1595	0.90	0.88, 0.91	<0.001			
Age relative *n* = 210/1121	0.99	0.98, 1.00	0.088	1.00	0.99, 1.01	0.671
Education *n* = 222/1465	1.19	1.13, 1.25	<0.001	1.13	1.07, 1.18	<0.001
% living with someone *n* = 224/1519	2.16	1.62, 2.87	<0.001	1.45	1.07, 1.97	0.016
Diagnosis *n* = 226/1595						
SCI/ not dementia	Ref			Ref		
MCI	0.49	0.22, 1.11	0.086	0.75	0.33, 1.72	0.503
Dementia	0.09	0.04, 0.18	<0.001	0.17	0.08, 0.36	<0.001
MMSE *n* = 223/1565	1.10	1.07, 1.14	<0.001	1.07	1.04, 1.10	<0.001
Clock drawing test *n* = 218/1538	2.49	1.84, 3.35	<0.001	1.60	1.17, 2.19	0.004
IQCODE *n* = 213/1395	0.36	0.27, 0.47	<0.001	0.52	0.39, 0.68	<0.001
PSMS *n* = 214/1344	1.40	1.29, 1.53	<0.001	1.25	1.14, 1.38	<0.001
IADL *n* = 186/1175	1.41	1.31, 1.53	<0.001	1.29	1.18, 1.41	<0.001
CSDD *n* = 191/1281	0.96	0.93, 0.98	<0.001	0.95	0.93, 0.98	<0.001
NPI-Q – psychosis	0.82	0.73, 0.92	0.001	0.87	0.78, 0.99	0.028
– affective	0.96	0.92, 1.01	0.084	0.95	0.91, 0.99	0.042
– agitation *n* = 156/1337*	0.94	0.87, 1.01	0.097	0.92	0.85, 0.99	0.040
RSS *n* = 204/1294	0.99	0.98, 1.00	0.143	0.99	0.98, 1.01	0.315

Estimated in logistic regression, crude and adjusted by age.

OR: odds ratio; CI: confidence interval; SCI: subjective cognitive impairment; MCI: mild cognitive impairment; MMSE: mini mental status examination; IQCODE: informant questionnaire on cognitive decline in the elderly – mean score of 16 items; PSMS: Physical Self Maintenance Scale; IADL: Instrumental Activities of Daily Living; CSDD: Cornell Scale for Depression in Dementia; NPI-Q: Neuropsychiatric Inventory-Questionnaire; RSS: Relatives’ Stress scale. *N is different for the three subsyndromes of NPI-Q; this is the lowest n.

**Table 3.** Odds ratios for being assessed for cognitive decline either in specialist healthcare (SpecCare), geriatric and old-age psychiatry clinics (‘other’) or SpecCare memory clinics versus in primary care (PrimCare) as reference category.

*N* = PrimCare/ SpecCare- ‘other’/ SpecCare-memory clinic	SpecCare – ‘Other’ (*n* = 967) vs PrimCare (*n* = 226) – unadjusted			SpecCare – ‘Other’ vs PrimCare adjusted for age			SpecCare – memory clinics (*n* = 628) vs PrimCare (*n* = 226) – unadjusted			SpecCare – memory clinics vs PrimCare adjusted for age		
	OR	95% CI	*p* Value	OR	95% CI	*p* Value	OR	95% CI	*p* Value	OR	95% CI	*p* Value
Gender (female = Ref)	1.06	0.79, 1.42	0.698	0.92	0.68, 1.25	0.606	1.47	1.08, 2.01	0.014	1.17	0.83, 1.64	0.370
Age	0.92	0.90, 0.94	<0.001				0.85	0.83, 0,87	<0.001			
Age relative *n* = 210/ 621/ 500	0.99	0.98, 1.01	0.324	1.00	0.99, 1.01	0.970	0.99	0.98, 0.99	0.021	1.01	1.00, 1.02	0.140
Education *n* = 222/ 858/ 607	1.14	1.08, 1.20	<0.001	1.11	1.05, 1.17	<0.001	1.26	1.20, 1.33	<0.001	1.17	1.11, 1.24	<0.001
Living with someone *n* = 224/ 916/ 603	2.02	1.50, 2.72	<0.001	1.52	1.11, 2.07	0.009	2.40	1.75, 3.28	<0.001	1.35	0.95, 1.91	0.093
Diagnosis												
SCI/ not dementia	Ref			Ref			Ref			Ref		
MCI	0.82	0.36, 1.88	0.64	0.98	0.42, 2.25	0.96	0.29	0.13, 0.65	0.003	0.48	0.21, 1.13	0.093
Dementia	0.16	0.08, 0.33	<0.001	0.22	0.10, 0.46	<0.001	0.05	0.02, 0.10	<0.001	0.12	0.05, 0.25	<0.001
MMSE *n* = 223/ 951/ 614	1.06	1.03, 1.10	<0.001	1.05	1.02, 1.09	0.002	1.20	1.16, 1.25	<0.001	1.13	1.09, 1.18	<0.001
Clock drawing test *n* = 218/ 931/ 607	0.55	0.41, 0.75	<0.001	0.70	0.51, 0.97	0.031	0.24	0.17, 0.33	<0.001	0.46	0.32, 0.66	<0.001
IQCODE *n* = 213/ 863/ 532	0.46	0.35, 0.61	<0.001	0.57	0.43, 0.76	<0.001	0.22	0.16, 0.30	<0.001	0.38	0.27, 0.52	<0.001
PSMS *n* = 214/ 826/ 518	1.26	1.15, 1.37	<0.001	1.19	1.08, 1.31	<0.001	1.88	1.67, 2.11	<0.001	1.49	1.32, 1.68	<0.001
IADL *n* = 186/ 709/ 466	1.29	1.19, 1.40	<0.001	1.23	1.13, 1.35	<0.001	1.72	1.56, 1.89	<0.001	1.46	1.32, 1.62	<0.001
CSDD *n* = 191/ 772/ 509	0.96	0.93, 0.98	0.002	0.96	0.93, 0.98	0.001	0.94	0.92, 0.98	<0.001	0.94	0.91, 0.97	<0.001
NPI-Q – psychosis	0.84	0.74, 0.94	0.004	0.87	0.77, 0.99	0.031	0.78	0.69, 0.89	<0.001	0.87	0.75, 1.01	0.065
– affective	0.96	0.92, 1.01	0.125	0.96	0.91, 1.00	0.067	0.96	0.91, 1.00	0.073	0.94	0.89, 0.99	0.029
– agitation *n* = 156/ 827/ 510*	0.94	0.87, 1.02	0.114	0.93	0.85, 1.00	0.059	0.94	0.86, 1.02	0.125	0.91	0.83, 0.99	0.039
RSS												
*n* = 204/ 798/ 496	1.00	0.98, 1.01	0.598	1.00	0.98, 1.01	0.636	0.98	0.97, 0.99	0.007	0.98	0.97, 0.99	0.035

Estimated in multinomial logistic regression, crude and adjusted by age.

OR: odds ratio; CI: confidence interval; SCI: subjective cognitive impairment; MCI: mild cognitive impairment; MMSE: mini mental status examination; IQCODE: informant questionnaire on cognitive decline in the elderly – mean score of 16 items; PSMS: Physical Self Maintenance Scale; IADL: Instrumental Activities of Daily Living; CSDD: Cornell Scale for Depression in Dementia; NPI-Q: Neuropsychiatric Inventory-Questionnaire; RSS: Relatives’ Stress scale.

*N is different for the three subsyndromes of NPI-Q; this is the lowest n.

### Factors associated with depression

Female gender, older age, being assessed in primary care, cognitive decline compared to 10 years earlier (IQCODE), and higher caregiver burden were associated with depression in patients (Table 4). Further, poorer cognition as assessed by the MMSE was associated with depression in unadjusted analyses (OR 0.98, CI 0.95, 0.99), but as seen in Table 4, the direction of the OR changed in the adjusted model to 1.04 (CI 1.00, 1.08), and the association was no longer significant. However, confounding effects of IQCODE and RSS on MMSE were observed. Further, the contribution on the model of the variables gender, living situation, IQCODE, and PSMS changed, in that their OR changed by 20% or more without changing direction, when caregiver burden (RSS) was entered in the model according to the predefined step. However, confounding effects were observed between RSS and the mentioned variables; caregivers scored themselves as having a higher burden when the patient was male, living with the caregiver, less educated, had more cognitive decline and dementia, and had more limitations in ADL.

**Table 4.** Odds ratios for having depressive symptoms (Cornell scale for depression in dementia), estimated in logistic regression, *N* = 1149.

	Unadjusted			Fully adjusted for all included variables in table		
	OR	95% CI	*p* Value	OR	95% CI	*p* Value
Gender (female = Ref)	0.87	0.69, 1.10	0.255	0.68	0.51, 0.91	0.009
Age	1.00	0.99, 1.01	0.617	0.97	0.96, 0.99	0.001
Education	0.98	0.95, 1.01	0.187	1.00	0.96, 1.04	0.888
Living with someone	0.84	0.66, 1.06	0.147	0.79	0.59, 1.07	0.134
Diagnosis						
MCI vs SCI/ not dementia	1.12	0.78, 1.61	0.531	1.17	0.76, 1.80	0.471
Dementia vs SCI/not dementia	1.56	1.12, 2.16	0.008	0.90	0.57, 1.42	0.654
MMSE	0.98	0.95, 0.99	0.045	1.03	0.996, 1.07	0.084
IQCODE	3.21	2.55, 4.04	<0.001	1.65	1.16, 2.33	0.005
PSMS	0.69	0.63, 0.75	<0.001	0.92	0.82, 1.03	0.153
RSS	1.11	1.09, 1.12	<0.001	1.10	1.08, 1.12	<0.001
Place of assessment (specialist healthcare = Ref)	1.41	1.01, 1.95	0.041	1.53	1.02, 2.30	0.039

OR: odds ratio; CI: confidence interval; SCI: subjective cognitive impairment; MCI: mild cognitive impairment; MMSE: mini mental status examination; IQCODE: informant questionnaire on cognitive decline in the elderly – mean score of 16 items; PSMS: Physical Self Maintenance Scale; RSS: Relatives’ Stress scale.

## Discussion

We found that patients diagnosed in primary healthcare were older, less educated, had poorer cognition and more limitations in ADL, had more BPSD, more depressive symptoms, more often lived alone, and were diagnosed with dementia more often compared to patients diagnosed in specialist healthcare. As young age is the main criterion for assessment in specialist healthcare, the younger age for the SpecCare cohort was expected. However, the lower educational level and larger percentage of single households in the PrimCare cohort were not due to the older age in this group.

The reason why patients in the PrimCare cohort were more often diagnosed with dementia may be because they were older and less educated. More often living alone adds to the likelihood of being diagnosed at a later stage in the course of the dementia syndrome [24]. The high proportion of SCI and MCI diagnoses in SpecCare, especially in the memory clinics, may indicate that these are patients with complicated symptoms or who seek assessment in a very early stage of cognitive decline and that it is not (yet) possible to conclude if it is dementia. It may also indicate that the referral criteria for assessment in specialist healthcare should not allow for patients with modest symptoms. The MMSE median score was 22 in the PrimCare cohort compared to 25 in the SpecCare cohort. In comparison, a study from 2010 comparing AD patients in memory clinics across Europe found that the mean MMSE score varied between 19.8 and 21.6 depending on region [25].

People living alone seem to have less access to specialist healthcare, as living alone is more frequent in the PrimCare cohort compared to the SpecCare cohort. This is in line with previous studies [26] and could be because co-resident relatives act as facilitators to access such services.

The recommendation in the Norwegian national guideline regarding assessment and diagnosis of people with symptoms of cognitive decline is that people older than age 65 and without complicated or unclear symptoms should be assessed and diagnosed by primary care. The difference in age found in this study, and the fact that the researchers giving the PrimCare patients research diagnoses for use in the study found that a large majority of the PrimCare patients had dementia, may indicate that the recommendations were followed. However, without data on comorbidity we cannot tell if the complicated cases were indeed assessed by specialist healthcare. A majority of the PrimCare patients received a diagnosis from their GP; explanations for no diagnosis could be no dementia and that the GP had not yet concluded the work-up when data were retrieved.

It may however also be that the GP lacks the knowledge or confidence to conclude on a dementia diagnosis. Giving a dementia diagnosis, including an aetiological diagnosis, is vital in order to provide the right treatment and post-diagnostic support. The finding that as many as 52 of 226 of the PrimCare patients were not given a diagnosis raises concerns regarding the knowledge of Norwegian GPs to correctly and sufficiently diagnose dementia. Even though the ICPC-2 diagnostic system does not require an aetiological diagnosis, GPs are encouraged to give such diagnoses. In cases where GPs are unable to conclude on a diagnosis, e.g. in patients with complicated symptoms and/or high comorbidity, the National dementia guideline recommends a referral to specialist healthcare, regardless of age. This is frequently done and is the reason why we did not use SpecCare data that was newer than the PrimCare data. We do not have information on how many of the 52 undiagnosed PrimCare patients were referred to specialist healthcare for a conclusion on diagnosis.

Our findings of more severe symptoms in patients in the PrimCare cohort, may be an argument that some of these PrimCare patients should have been referred to specialised healthcare, as several symptoms could be better assessed there. It is important to stress that according to the guideline, anyone presenting complicating factors such as comorbidity or neuropsychiatric symptoms should be referred to specialist healthcare – regardless of age. Comorbidity increases with age, and age may therefore be an argument for assessment in specialist healthcare rather than against. There are clearly advantages of assessing patients with cognitive decline in specialist healthcare; the (usually) higher level of dementia-specific knowledge, including ability to assess comorbidity as well as better diagnosing being some of them. There are however also advantages of assessing patients in primary healthcare. As assessments in primary healthcare are usually done in the patients’ home, the patients may be less stressed, and health care personnel can observe e.g. functional ability in the patients’ own environment. Also, assessments can be done over time, and issues found in the assessment can be addressed immediately without a transition. It is an advantage that assessment, diagnosis and post-diagnostic support is done by the same few people. The GP usually knows the patient well and is therefore well suited to guide the patient after the diagnosis is given, health care personnel will after the assessment have first-hand information and will have already started forming an alliance. It may also be argued that the more severe symptoms in the PrimCare cohort represent more severe dementia rather than the patients being complicated to diagnose.

The patients from the geriatric and old-age psychiatry outpatient clinics were more comparable to the PrimCare patients than patients in the memory clinics were. This indicates that SpecCare is a heterogeneous cohort. It is often clear that a patient under 65 years with cognitive decline should be referred to a memory clinic, but it is less clear whether patients over 65 years with cognitive decline should be assessed by their GP or referred to specialist healthcare and to which type of outpatient clinic. It may be that patients in the latter group were diagnosed in specialist healthcare even though the GP could have done it. Factors such as geographic location/availability of specialist healthcare and the individual GP’s confidence in assessing symptoms of cognitive decline may also play a role in where people are diagnosed.

Our findings underline the importance of post-diagnostic support. People diagnosed with dementia in primary healthcare need services tailored to their needs and reduced functioning. We suggest that service providers pay special attention to the relatively high presence of depression (CSDD median 7, IQR 3, 12), the limitations in ADL (PSMS median 4, IQR 3, 5), and the finding that these patients often live alone without daily supervision by a relative. People with dementia living alone are more isolated, and previous studies have found that they have more unmet needs than those living with others, which makes them a vulnerable and high-risk group [27].

In addition to depression being more prevalent in the primary healthcare cohort, our findings indicate that depression was associated with female gender, older age, and greater decline in cognitive functioning. Caregiver burden was also strongly associated with patients’ depression which is in line with earlier studies [28]. This association might be because the patient’s depression leads to higher caregiver burden. However, as the caregiver completes the depression scale in our study, it may also be that caregivers who experience high burden report more symptoms of depression in the patient.

The triad of late-life depression, cognitive impairment, and disability is complex. Depression promotes disability; disability fosters depression; and cognitive impairment complicates this relationship by influencing both disability and depression [29]. This complexity should be considered when tailoring post-diagnostic support for people diagnosed with dementia in primary healthcare. Poorer cognition and reduced performance in ADL among the PrimCare cohort may have led to less engagement in pleasant activities. According to behavioural models, depressive symptoms may be intensified or maintained by the absence of positive feelings resulting from participation in enjoyable and meaningful activities [30]. Individual and group interventions targeting activities, such as behavioural activation and Cognitive Stimulation Therapy (CST) have been found to reduce depressive symptoms and improve scores on ADL of community-dwelling older people [30,31]. A review by Nyman *et al.* (2016) highlights that providing activities for people with dementia goes beyond mere pleasure to meeting fundamental psychosocial needs [4]. The Norwegian national guideline on dementia strongly recommends psychosocial interventions based on the interests, preferences, and functional level of the person with dementia [9].

### Strengths and limitations

The study’s strengths are the large number of patients included and the use of standardised measures by experienced health personnel.

Its limitations are as follows: (1) the data are from 2011–2014, which may result in poorer generalisability today; (2) lack of comparable measures of comorbidity which makes it hard to say if the complicated cases have been handled by specialist healthcare; (3) the large number of municipalities and outpatient clinics represented, with a risk of data collectors using the instruments differently; (4) research diagnoses were used for all PrimCare participants relying only on data available and not considering other information of importance for the diagnoses; (5) 4% of the participants in the PrimCare cohort and 10% in the SpecCare cohort had missing data on all the proxy-based measures; (6) aetiological diagnoses were not used in this study; (7) causality has not been studied.

## Conclusion

People assessed for cognitive decline in primary healthcare were older, less educated, had poorer cognitive functioning and more limitations in ADL, had more BPSD and more depressive symptoms, were more likely to live alone, and were more often diagnosed with dementia than people assessed in specialist healthcare.

The relatively high presence of depression and ADL limitations of people assessed in primary healthcare, as well as the finding that they more often lived alone, present important facts to consider when planning and providing post-diagnostic support for this group.
